# *Francisella *RNA polymerase contains a heterodimer of non-identical α subunits

**DOI:** 10.1186/1471-2199-12-50

**Published:** 2011-11-22

**Authors:** Damir Mukhamedyarov, Kira S Makarova, Konstantin Severinov, Konstantin Kuznedelov

**Affiliations:** 1 Department of Biochemistry and Molecular Biology and Waksman Institute of Microbiology, Rutgers, the State University of New Jersey, Piscataway, NJ 08854, USA; 2 National Center for Biotechnology Information, National Library of Medicine, National Institutes of Health, 8600 Rockville Pike, Bethesda, Maryland 20894, USA; 3 Institutes of Gene Biology and Molecular Genetics, Russian Academy of Sciences, Moscow, Russia

## Abstract

**Background:**

All sequenced genomes of representatives of the *Francisella *genus contain two *rpoA *genes, which encode non-identical RNA polymerase (RNAP) subunits, α1 and α2. In all other bacteria studied to date, a dimer of identical α subunits initiates the assembly of the catalytically proficient RNAP core (subunit composition α_2_ββ'). Based on an observation that both α1 and α2 are incorporated into *Francisella *RNAP, Charity *et al. *(2007) previously suggested that up to four different species of RNAP core enzyme might form in the same *Francisella *cell.

**Results:**

By *in vitro *assembly from fully denatured state, we determined that both *Francisella *α subunits are required for efficient dimerization; no homodimer formation was detected. Bacterial two-hybrid system analysis likewise indicated strong interactions between the α1 and α2 N-terminal domains (NTDs, responsible for dimerization). NTDs of α2 did not interact detectably, while weak interaction between α1 NTDs was observed. This weak homotypic interaction may explain low-level transcription activity observed in *in vitro *RNAP reconstitution reactions containing *Francisella *large subunits (β', β) and α1. No activity was observed with RNAP reconstitution reactions containing α2, while robust transcription activity was detected in reactions containing α1 and α2. Phylogenetic analysis based on RpoA resulted in a tree compatible with standard bacterial taxonomy with both *Francisella *RpoA branches positioned within γ-proteobacteria. The observed phylogeny and analysis of constrained trees are compatible with *Francisella *lineage-specific *rpoA *duplication followed by acceleration of evolutionary rate and subfunctionalization.

**Conclusions:**

The results strongly suggest that most *Francisella *RNAP contains α heterodimer with a minor subfraction possibly containing α1 homodimer. Comparative sequence analysis suggests that this heterodimer is oriented, in a sense that only one monomer, α1, interacts with the β subunit during the α_2_β RNAP subassembly formation. Most likely the two *rpoA *copies in *Francisella *have emerged through a lineage-specific duplication followed by subfunctionalization of interacting paralogs.

## Background

Bioinformatics analysis reveals that two paralogous *rpoA *genes, each encoding non-identical proteins homologous to bacterial RNA polymerase (RNAP) α subunits, are present in the genome of *Francisella tularensis *[1]. The bacterial RNAP core enzyme has subunit composition α_2_ββ'. Variations including fusion of the largest subunits, β and β', in *Helicobacter *and *Wolinella *genera [2,3], and split the largest subunit in some cyanobacteria [4] have been reported, but overall, the subunit composition of RNAP core is conserved. The α subunit homodimer initiates bacterial RNAP assembly. The α subunit monomers dimerize through their N-terminal domain (NTD) [5,6]. The C-terminal domain (CTD) is connected to NTD through a flexible tether [7]. The αCTD is not required for assembly but is involved in transcriptional regulation [8-10]. The αNTD homodimer provides a platform for interaction with the two large RNAP subunits [11,12]. Determinants in α important for interactions with β and β' subunits have been localized by mutagenesis and hydroxyl-radical footprinting studies [5-8,13-15]. Substitutions at positions 45 and 48 of *Escherichia coli *α subunit completely (R45A) or partially (L48A) prevented formation of the α_2_β RNAP subassembly [16]. Two point substitutions at positions 86 and 173, and two-amino-acid insertions at positions 180 and 200 of *E. coli *α caused defects in β' binding without affecting the α_2_β assembly formation [16,17]. RNAP containing oriented *E. coli *α heterodimers have been prepared both *in vitro*, by reconstitution from recombinant subunits, and *in vivo*, by co-expression of genes for recombinant subunits, by using one α subunit lacking the R45A substitution and one α subunit having the R45A substitution [18,19]. Functional analysis of RNAP containing oriented α heterodimers confirmed that asymmetrical arrangement of α leads to non-identical functions of each monomer in transcription regulation [18,19].

RNAP core enzymes from archaea and eukaryotes contain homologs of each of the bacterial RNAP core subunits. However, rather than having two identical α subunit homologs, they contain two different α-like polypeptides (RPB3 and RPB11 in the case of eukaryotic RNAP II) that form a heterodimer, which serves as a platform for RNAP assembly [20].

The presence of two different genes (*rpoΑ1 *and *rpoΑ2*) in the genome of *Francisella *suggests that up to four RNAP core enzymes differing in subunit composition could be present in the cells: two enzymes containing α homodimers, (α1)_2_ββ' and (α2)_2_ββ', and two enzymes containing α heterodimers, (α1α2)ββ' and (α2α1)ββ' [1]. The heterodimers could differ from one another with respect to which α interacts with the β subunit of RNAP and which α interacts with β' [18,19]. Promoter recognition properties of RNAP holoenzymes formed from these different core enzyme molecules may differ, since CTD of α1 and α2 may be capable of different protein-DNA and protein-protein interactions during transcription initiation [18,19]. Further, if holoenzymes containing RNAP core enzymes of different composition indeed respond differently to transcription factors and elements, then *F. tularensis *may regulate the spectrum of expressed genes by altering the relative ratio of core enzymes with different α subunit composition, which would be a novel paradigm of transcription regulation in bacteria.

Evidence of that both α1 and α2 subunits are incorporated into *F. tularensis *RNAP has been reported earlier by Charity *et al. *[1]. These authors demonstrated that RNAP affinity purified from *F. tularensis *strain expressing the β' subunit with fused TAP-tag contained both α1 and α2. These experiments clearly show that both *rpoA *genes are active and their products are components of RNAP but do not inform about the actual subunit composition of *F. tularensis *RNAP. Based on predicted dimerization determinants in other bacteria [21], Charity *et al. *hypothesized that α1 and α2 might exclusively form either homodimers or heterodimers [1]. In the present study, we describe the results of *in vitro *analysis of assembly of RNAP from *F. tularensis *subspecies *novicida*. Our results indicate that RNAP core containing an α heterodimer is the main, perhaps the only, species of RNAP in this organism. We further present results of phylogenetic analysis that provide a plausible scenario for the appearance of two paralogous *rpoA *genes in the *Francisella *lineage.

## Results

### *F. tularensis *α heterodimer but not homodimers efficiently assembles *in vitro*

**Figure 1:**
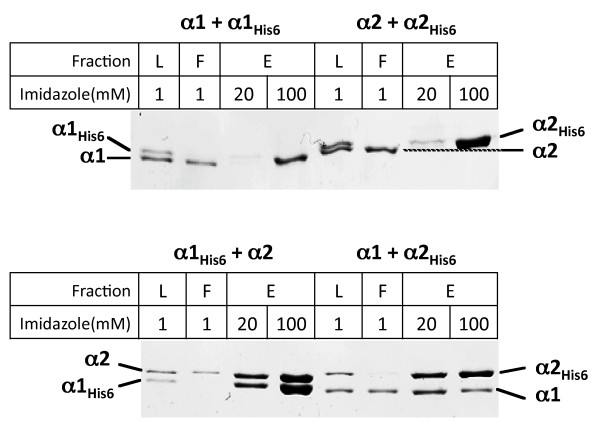
***In vitro *assembly of *Francisella *α homo- and heterodimers**. Reactions containing indicated proteins were combined at denaturing conditions and, following dialysis into a buffer favouring RNAP assembly, were fractionated using Ni^2+^-affinity chromatography. Coomassie-stained SDS gels are presented. "L" - load, "F" - flow-through, "E" - elution with buffers with indicated concentration of imidazole.

To experimentally address the ability of *F. tularensis *RNAP α subunits to form homo- and heterodimers, we investigated the ability of recombinant *F. tularensis *α subunit proteins with C-terminal His_6_-tags to pull down untagged counterparts during ion metal affinity chromatography. As shown previously, *F. tularensis *RNAP α subunits have different electrophoretic mobilities, with α1 migrating significantly faster than α2 [1]. In addition, His_6_-tags alter electrophoretic mobility of both α1 and α2 enough to separate tagged and untagged α subunits of the same kind (Figure 1).

Therefore, because each of the four proteins used in the pull-down assay has a characteristic electrophoretic mobility, it is possible to detect the efficiency of both hetero- and homodimer formation. Various pairwise combinations of α subunits were mixed at denaturing conditions (6 M guanidinium chloride), the denaturing reagent was removed by dialysis at conditions favouring bacterial RNAP assembly from isolated subunits [22], and reconstitution reactions (labelled "L" on Figure 1) were loaded on Ni^2+^-affinity columns. Flow-through (F) was collected and retained protein was eluted (E) with different concentrations of imidazole in the buffer. Aliquots of each fraction were next analyzed by SDS-PAGE. As can be seen from Figure 1 (top panel), no co-immobilization of untagged α subunit in reactions that contained tagged and untagged versions of subunit of the same kind was detected. In contrast, heterodimers were readily detected when either α1His_6 _or α2His_6 _were used as "baits" for co-immobilization of, respectively, α2 or α1 (Figure 1, bottom panel). We conclude that *F. tularensis *RNAP α subunits do not appreciably form homodimers, at least at the conditions of *in vitro *RNAP assembly.

### α1NTD and α2NTD efficiently interact in bacterial 2-hybrid system

RNAP α subunit is a two-domain protein, with its N-terminal domain being primarily responsible for dimerization and interaction with large RNAP subunits, while the C-terminal domain, CTD, which is connected to NTD through a flexible linker, is primarily responsible for interactions with transcription factors and DNA upstream of the -35 promoter element [23]. Weak dimerization of isolated αCTD has been reported and may be of regulatory significance [7]. To independently study dimerization of various domains of *F. tularensis *α subunits, we used the bacterial two-hybrid system [24]. Eight two-hybrid plasmids expressing bait and prey fusions of each α domain were constructed and 16 pairwise combinations were tested. The results are presented in Table 1. As can be seen, in agreement with *in vitro *co-immobilization data, strong interactions between αNTDs of different kinds were detected. αCTDs did not appreciably interact with each other or with αNTDs. The level of homotypic interaction between α1NTD was above the background, potentially indicating formation of α1NTD homodimer, while the level of α2 homodimer formation was at the background level.

**Table 1:** Bacterial two-hybrid analysis of interactions between domains of α1 and α2 subunits of *F. tularensis *RNAP

pBRαLn+pACλcI	α1NTD	α1CTD	α2NTD	α2CTD
**α1NTD**	171 ± 28	44 ± 1	**1412 ± 323**	53 ± 9

**α1CTD**	110 ± 13	95 ± 8	133 ± 5	99 ± 16

**α2NTD**	**662 ± 26**	47 ± 9	74 ± 4	49 ± 3

**α2CTD**	102 ± 3	101 ± 1	112 ± 6	86 ± 9

Eight two-hybrid plasmids expressing bait (1^st ^column) and prey (1^st ^row) fusions of each α subunit domain were constructed and 16 pairwise combinations were tested in a reporter strain by measuring β-galactosidase activity (in Miller units). Each combination was tested at least three times independently. Mean and standard deviation values are presented. Three kinds of measurements were taken to determine background levels of β-galactosidase activity, which was found to be (in Miller units) 111 ± 26 in host reporter cells with no plasmids, 110 ± 9 in cells transformed with pBRαLN-α1NTD only, and 50 ± 7 in cells transformed with pACλcI-α1NTD only.

### Formation of the α_2_β subassembly *in vitro*

**Figure 2:**
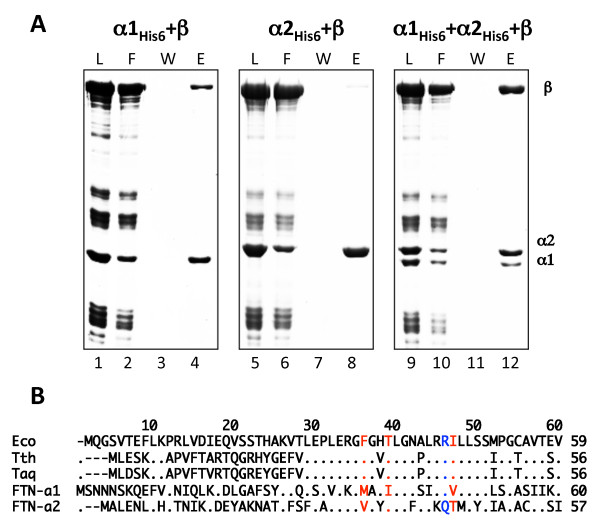
***In vitro *assembly of *Francisella *α**_**2**_**β RNAP subassembly**. **A**. Reactions were assembled and analyzed as described in Figure 1 legend. "W" -wash with excess of loading buffer. Proteins were eluted with a buffer containing 100 mM imidazole. **B**. Sequence alignment of the α subunit segment involved in dimerization and interaction with the β subunit. A segment of *E. coli *α subunit (amino acids 1-59) is shown at the top (single-letter amino acid code). Corresponding sequences from *Thermus aquaticus *(Taq), *Thermus thermophilus *(Tth), and *Francisella tularensis *RpoA variants are aligned below. Dots indicate identities, hyphens - gaps. Amino acids highlighted in red form a cluster important for α homodimer formation in *E. coli*. Amino acid highlighted in blue is responsible for the interaction with β.

RNAP assembly follows a conserved pathway, whereby the β subunit interacts with the α dimer, leading to the formation of α_2_β - a stable intermediate of RNAP assembly that can be observed both *in vivo *and *in vitro *[11,16]. We performed *in vitro *RNAP assembly using His_6_-tagged *F. tularensis *α subunits and untagged recombinant *F. tularensis *β. The results indicated that β was most efficiently immobilized when both α subunits were present in the assembly reaction (Figure 2A, lane 12). Only trace amounts of β were co-immobilized in reactions containing α2 (Figure 2A, lane 8) and thus likely represented non-specific binding (note that an excess of α2 was used in this reaction). The amount of β co-immobilized in reactions containing α1 (lane 4) was higher than the background but clearly less than that observed in reactions containing both α subunits. We conclude from these experiments that β interacts most efficiently with α heterodimer. Detected interaction between β and α1 can proceed through α1 monomer or, alternatively, the β subunit may stimulate formation of the α1 homodimer.

### *In vitro *transcription by recombinant *F. tularensis *RNAP

**Figure 3:**
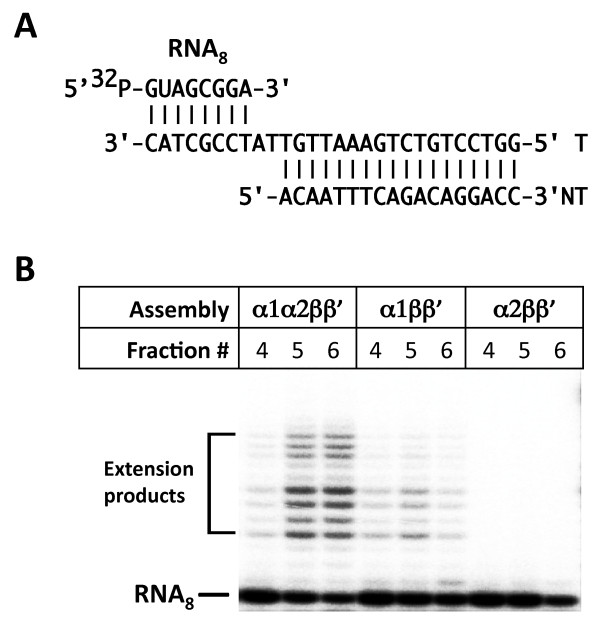
**Transcription activity of *in vitro *assembled *Francisella *RNAP core enzymes**. **A**. The structure of nucleic acid scaffold used to test assembled enzymes activities is schematically shown. **B**. Superose 6 fractions obtained after separation of *in vitro *RNAP assembly reactions containing indicated subunits were combined with nucleic acid scaffold containing a radioactively labelled RNA primer and reactions were supplemented with NTP. Reaction products were analyzed by denaturing PAGE. An autoradiograph is presented.

To validate data obtained using two-hybrid analysis and α dimer/α_2_β RNAP subassembly *in vitro *reconstitution, *in vitro *RNAP assembly and transcription experiments were performed. Three *in vitro *RNAP assembly reactions contained recombinant *F. tularensis *β and β' subunits and α1, α2, or both α1 and α2 (the ω subunit was omitted from assembly reactions as it is not essential for RNAP basic function [25,26]). Assembled RNAP reactions were passed through a gel-filtration column, fractions that eluted at retention times expected for RNAP core elution were collected and tested for transcription activity on a nucleic acid scaffold shown in Figure 3A. Nucleic acid scaffolds mimic the conformation of nucleic acids in transcription elongation complexes. RNAP complexes with nucleic acids scaffolds are catalytically active and serve as a convenient tool to study transcription elongation properties of the enzyme [27]. Reactions were combined with NTP, and elongation of radioactively labelled 8-nt RNA component of the scaffold ("RNA_8_") followed. The results are presented in Figure 3B. As can be seen, most efficient elongation of the RNA primer was observed in fractions obtained from RNAP assembly reaction containing both α subunits. Fractions of RNAP assembly reaction that contained α2 only were completely inactive. Fractions of RNAP assembly reaction containing α1 only demonstrated low but detectable transcription activity. We therefore conclude that *F. tularensis *RNAP assembles efficiently when both kinds of α subunits are present; α2 alone is unable to promote RNAP assembly; α1 alone supports RNAP assembly, albeit with low efficiency, possibly due to low level of α1 homodimer formation.

### Evolution of RpoA in *Francisella*

To gain insight into the evolution of two paralogs of RpoA in *Francisella*, we retrieved all RpoA sequences in all of the 1055 completely sequenced bacterial genomes available in the RefSeq database. We found that in addition to *Francisella*, two *rpoA *genes (*rpoA1 *and *rpoA2*) are present in several other genomes, namely in three *Chloroflexus *species (*C. aggregans *DSM 9485; *C. aurantiacus *J-10-fl; *C. *sp. Y-400-fl), in *Streptomyces avermitilis *MA-4680, in *Psychromonas ingrahamii *37, and in *Leptospira borgpetersenii *serovar Hardjo bovis L550 (see also Additional file 1). In the latter two cases the two *rpoA *copies are identical and are apparently the result of very recent genome segment duplications that also include a number of other genes.

**Figure 4:**
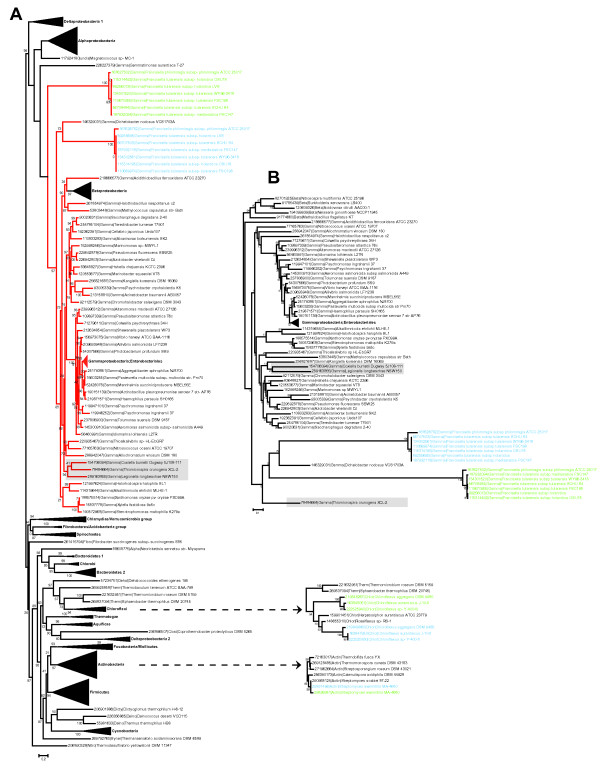
**Phylogenetic trees of the bacterial RpoA**. **A**. A set of 368 RpoA sequences from 355 representative genomes was aligned using MUSCLE program. The Maximum likelihood tree with bootstrap values applied was built for 246 informative aligned positions using FastTree program. **B**. A set of 84 RpoA sequences from 76 representative genomes of γ- and β-proteobacteria was aligned using MUSCLE program. The Maximum likelihood tree with bootstrap values applied was built for 296 aligned informative positions using RAxML program. The evolutionary model for tree reconstruction (WAG with gamma-distributed evolutionary rates) has been selected using ProtTest program. Colour code: light blue - RpoA1 subunits encoded in ribosomal operons, the default location for all bacteria; light green - RpoA2 subunits encoded elsewhere in the genome. Dashed arrows point to subtrees with two other (non-*Francisella*) instances of RpoA duplication: *Chloroflexus *species and *Streptomyces avermitilis*. Each terminal tree node is labelled with GenBank Identifier (GI) number, five-letter taxonomy code and full systematic name of an organism. The taxonomy code is the following: Gamma - γ-proteobacteria; Beta - β-proteobacteria; Alpha -α- proteobacteria; Clost - *Clostridia*; Dicty - *Dictyoglomi*; Deino -*Deinococcus/Thermus *group; Syner - *Synergistetes*; Nitro - *Nitrospirae*; Therm -*Thermomicrobia*; Dehal - *Dehalococcoidetes*; Fibro - *Fibrobacteres/Acidobacteria *group; Gemma - *Gemmatimonadetes*; uncla - unclassified. Compressed branches (black triangles) are labelled according to taxonomy. Branches leading to γ-proteobacteria are red. The shaded clade indicates close relatives of *Fransicella *according to taxonomy based on 16S rRNA and concatenated ribosomal proteins phylogeny.

In order to address an alternative possibility, that one of the RpoA paralogs in *Francisella *could have been horizontally transferred from a distant bacterial (other than γ-proteobacteria to which the *Francisella *genus belongs) lineage instead of arising through gene duplication, we reconstructed RpoA phylogenetic tree for a representative set of bacteria including those that contain *rpoA *duplications listed above (Figure 4A). The resulting tree is generally very well compatible with bacterial taxonomy, which is not surprising considering the fact that RNAP subunits are among the best phylogenetic markers [28-30]. The position of both RpoA branches corresponding to *Francisella *within γ-proteobacteria is confidently supported by bootstrap analysis (bootstrap probability of 0.93). Thus, it is unlikely that any of the *Francisella rpoA *genes were transferred from outside of the γ-proteobacterial lineage. Branches leading to both *Francisella *RpoA proteins are extremely long, which might cause an artefact of the long-branch attraction, making the *Francisella *RpoA positioning unreliable. To test hypotheses for an alternative position of *Francisella *RpoA branches, we used RAxML [31] program to reconstruct a phylogenetic tree for γ -proteobacteria with β-proteobacteria outgroup (Figure 4B), made constrained trees and compared the maximum likelihood values for the best tree (Figure 4B) and constrained trees. The first constrained tree was designed to test a hypothesis of monophyly of two RpoA paralogs of *Francisella*; the second tree was designed to test a hypothesis of monophyly of both *Francisella *RpoA and of homologs from *Coxiella*, *Legionella*, and *Thiomicrospira *- species that are the closest taxonomic relatives of *Francisella *(Additional file 2). The analysis showed that none of the hypotheses could be rejected, suggesting that the positioning of RpoA at the root of γ-proteobacteria could be explained by long-branch attraction artefacts. Thus, we conclude that the two RpoA copies in *Francisella *most likely emerged through a duplication followed by acceleration of the evolutionary rates of both paralogs.

## Discussion

Representatives of the bacterial genus *Francisella *are unusual with respect to RNAP composition, in that they contain two different α subunits encoded by two paralogous genes *rpoΑ1 *and *rpoΑ2*. The presence of two different α subunits in affinity-purified RNAP preparations [1] suggested that as many as four different species of RNAP core enzyme could be present in the single cell. Here, we studied *Franicella *RNAP by means of *in vitro *assembly. Our results show that *Franicella *α heterodimer (α1α2) efficiently assembles *in vitro *from fully denatured state and homodimers are not detected. Bacterial two-hybrid analysis indicates that in addition to efficient α heterodimer assembly, some dimerization of α1 may also occur. Thus, the efficiency of α dimerization is clearly a major factor that should affect subunit composition of *Francisella *RNAP. As was determined from crystal structure analysis, the main structural elements of the α dimer interface of *E. coli *are two α-helices, H1 and H3, orthogonally oriented to each other [21]. These helices from one monomer participate in a coiled-coil-like interaction with their counterparts in the other monomer. Within these helices, Kannan et al. [32] identified a cluster of amino acids stabilizing interactions at the *E. coli *α dimer interface, with residues 35F, 38T, and 39L emanating from one α monomer and residues 46I, 50S, and 227Q from another. Of particular interest are three of them, namely, 35F, 38T, and 46I, point mutations at which partially (α-T38A) [16] or completely (α-F35A, α-I46S) [32] prevented the dimer formation. As can be seen from Figure 2B, amino acids at these positions are conserved (identical) between *E. coli *and *Thermus*, while amino acids in many of the corresponding positions in both α1 and α2 subunits from *Francisella *differ. Since these positions are critical for the dimer formation, it is reasonable to assume that some amino acids at these positions of *Francisella *α subunits, for example, α1-36M, α1-39I, α2-33V, and α2-47T, may be unfavourable to the assembly of homodimers. However, in the absence of crystal structure or systematic mutagenesis data, it is currently not possible to identify structural reasons for hetero- and homodimerization of *Francisella *α subunits.

During RNAP assembly in organisms where α subunit forms a homodimer, the β subunit is free to interact with either α monomer to form the α_2_β subassembly. The situation must be different in the case of *F. tularensis*, where α heterodimers form preferentially. In *E. coli*, an evolutionary conserved α subunit residue Arg^45 ^is critical for β subunit interaction with the α dimer [16,18,19]. In *F. tularensis*, the corresponding position in α1 also contains an arginine (Arg^46^), while in α2 this position is occupied by glutamine (Gln^42^; Figure 2B). Thus, it appears that β subunit in *F. tularensis *will be specifically interacting with the α heterodimer through α1. That α1 contains determinants for interactions with β also follows from results of the α_2_β subassembly reconstitution and *in vitro *transcription data, since *Francisella *RNAP containing α1 homodimer is functional and formation of an (α1)_2_β intermediate can be detected *in vitro*, albeit with low efficiency. The latter result suggests that β may stimulate α1 dimerization. An alternative possibility would be β interacting with one α1 monomer, followed by association with another α1 and β', or the α1β' complex. Be that as it may, our data suggest that bacteria of *Francisella *genus produce a major form of RNAP containing an oriented α1α2 heterodimer, and a minor form containing α1 homodimer.

As shown earlier by hydroxyl-radical-mediated proteolysis [14], the segments of *E. coli *α most strongly protected by β correspond to amino acids 30-55 and 65-75, and the segments of α most strongly protected by β' correspond to amino acids 175-185 and 195-210. Single alanine substitutions of *E. coli *α Lys^86 ^and Val^173 ^and two-amino-acid insertions at positions 180 and 200 of *E. coli *α cause defects in β' binding without affecting the α_2_β assembly formation [16,17]. To evaluate the ability of *Francisella *α1 and α2 subunits to interact with the β' subunit, we compared sequences of *E. coli *α subunit involved in interaction with β' [14,21] to those in *F. tularensis *α1 and α2 subunits (Additional file 1). The results reveal that a lysine at a position corresponding to *E. coli *α position 86 is present in both α polypeptides from *Francisella*, while amino acids corresponding to *E. coli *α Val^173 ^are, respectively, a valine and a leucine in α1 and α2. Similarly, the site of one two-amino-acid insertion that destroys β' interaction with α_2_β in *E. coli*, Val^180^, has as its counterpart a valine in α2 and an isoleucine in α1 of *Francisella*. These conservative changes are unlikely to affect the efficiency of β' binding by the α polypeptides. Interestingly, the site of the residue at the site of the second insertion affecting β' interaction with α_2_β in *E. coli*, Lys^200^, is conserved in *Francisella *α2 and α subunits from other bacteria (see Additional file 1), but is substituted with threonine in α1. The results thus implies that *Francisella *β' interacts with α2. Further experiments will be needed to prove this conjecture.

Our phylogenetic analysis indicates that none of the *Francisella rpoA *genes was transferred from outside of the y-proteobacterial lineage. In fact, both genes most likely emerged through duplication of an ancestral single gene followed by acceleration of evolutionary rate of both paralogs. Acceleration of *rpoA *evolutionary rate after the duplication apparently was accompanied by subfunctionalization of *Francisella *α subunits, ultimately leading to accumulation of substitutions in residues responsible for homodimerization and involved in the (β and β' subunit interaction.

Similar events, albeit on much longer time intervals, must have led to formation of two very different α-like subunits in eukaryotes and archaea. The large α-like subunit (RPB3 in eukaryal RNAP II, AC40 in RNAP I and RNAP III, Rpo3 (also known as RpoD) in archaea) heterodimerizes with its much smaller counterpart (RPB11 in eukaryal RNAP II, AC19 in RNAP I and RNAP III, Rpo11 (also known as RpoL) in archaea) [33-36]. Crystallographic and functional analyses indicates that large α homolog makes interaction with the second-largest (β-like) subunit through a surface that contains residue homologous to *E. coli *α Arg^45 ^[35,37], and is thus formally similar to *Francisella *α1. The smaller α homolog of eukaryal and archaeal RNAP thus corresponds to *Francisella *α2. One should not take this analogy to far though, since in eukaryotes and archaea, the α heterodimer is not sufficient for recruitment of large RNAP subunits in the complex. Eukaryal RPB10 and RPB12 and their archaeal homologs Rpo10 (also known as RpoN) and Rpo12 (also known as RpoP) form a stable complex with all four polypeptides playing an essential role in assembly and stability of the RNAP complex [36,38,39].

Evolution of the *rpoA *duplication presented here is one of the best demonstrations in support of the Lynch's subfunctionalization scenario where both copies are subject to relaxed selection and acceleration of the evolutionary rates but rarely develop a new or specialized function [40]. The fact that bacterial RNAP α subunit functions as a dimer should make it particularly prone to duplication/subfunctionalization. Indeed, while the impetus for our study came from an apparently unique situation with two different α subunits in *Francisella*, bioinformatics analysis revealed additional instances of *rpoA *duplications, some fairly recent, like in *S. avermitilis*, others more ancient, like in *Chloroflexus *species. Despite the fact that these RpoA paralogs are being ancestral for the *Chloroflexus *species, no drastic substitutions in regions responsible for dimerization and/or β/β' interactions have accumulated, suggesting that in contrast to the situation observed in *Fransicella*, the two α subunits of *Chloroflexus *may still be functionally equivalent. It is likely that many more instances of *rpoA *duplications and subfunctionalization will be found in the future.

## Conclusions

The data presented here support the following conclusions: (1) only *Francisella *α-heterodimer (α1α2) can be efficiently assembled *in vitro*; (2) strong direct interactions between α1NTD and α2NTD only have been detected in the bacterial two-hybrid system; (3) β interacts more efficiently when both of α1 and α2 presented in the reconstitution mix; (4) interaction between α1 and β subunits was observed to be stronger than interaction between α2 and β; (5) based on phylogenetic analysis, two *rpoA *copies in *Francisella *most likely must have emerged through a duplication followed by acceleration of the evolutionary rates of both paralogs.

## Methods

### Bacterial strains

*E. coli *NovaBlue Singles competent cells (Novagen) were used for initial cloning and plasmid propagation. *E. coli *BL21 (DE3) cells were used for protein overproduction. Reporter *E. coli *strain FW102 F'O_L_2-62 [41] was used for bacterial two-hybrid experiments.

### Cloning and expression

*Francisella tularensis novicida *genomic DNA has been provided by Dr. Michael Ibba (Ohio State University). Primers for PCR amplification *of rpo *genes were designed using *Francisella tularensis *subsp. *novicida *(FTN) strain U112 genome sequence data [GenBank: NC_008601]. The primers allowed cloning of amplified FTN *rpo *genes in pET series *E. coli *expression plasmids between the *NcoI *and *EcoRI *(or *XhoI *to express C-terminally hexahistidine-tagged α subunits) restriction sites. The plasmids pET28-FtnA1His, pET28-FtnA2His, pET28-FtnA1, pET28-FtnA2, and pET28-FtnB, pET30-FtnC overexpressing, respectively, C-terminally hexahistidine-tagged αlHis_6 _and α2His_6_, and untagged α1, α2, and β, and N-terminally hexahistidine-tagged β' subunits, were constructed using routine cloning methods and verified by sequencing of entire *rpo *portions for each plasmid. BL21 (DE3) cells harbouring the pET28a-rpo-gene plasmids were grown in 500 ml of LB medium, supplemented with 25 μg/ml kanamycin, at 37°C until an OD_600 _of around 1 was reached. Then the culture was induced to express an RNAP subunit by the addition of 1 mM isopropyl β-D-1-thiogalactopyranoside and allowed to grow for 2-4 hours. Cells were harvested by centrifugation and stored at -80°C before use.

### Protein purification

Frozen cells were thawed and lysed by sonication in a buffer containing 40 mM Tris-HCl, pH 8.0, 0.1 M NaCl, 10 mM EDTA, 10 mM 2-ME. The lysate was clarified by centrifugation for 30 min at 15,000 × g, and supernatant was supplemented with ammonium sulphate to precipitate soluble proteins (α2, α1His_6_, α2His_6_). Pellets containing insoluble proteins (α1, β, α1His_6_, α2His_6_, His_6_-β') were resuspended in 40 mM Tris-HCl, pH 8.0, 0.3 M KCl, 10 mM EDTA, 0.2% Na-deoxycholate to prepare inclusion bodies as described before [22,42]. Ammonium sulphate precipitate was collected by centrifugation, and the pellet, containing untagged protein (α2), was dissolved in 40 mM Tris-HCl, pH 8.0, 0.1 M NaCl, 1 mM EDTA, 2 mM 2-ME, 0.1 mM PMSF and loaded onto a 1 ml HiTrap Heparin HP column (GE Healthcare) equilibrated in the same buffer. The bound protein was eluted by a linear gradient of NaCl (from 0.1 to 1.0 M) in the same buffer. The pellet containing α1His_6 _or α2His_6 _was dissolved in 20 mM Tris-HCl, pH 8.0, 0.5 M NaCl, 2 mM 2-ME, 0.1 mM PMSF, 5 mM imidazole and loaded onto the 1 ml HiTrap Chelating HP column (GE Healthcare) charged with Ni^2+^. The column was washed and bound protein was step-eluted with 20, 50, or 100 mM imidazole. Fractions containing pure protein were pooled and dialyzed against two changes of 500 volumes of 40 mM Tris-HCl, pH 8.0, 100 mM NaCl, 0.5 mM EDTA, 0.5 mM 2-ME, 10% glycerol, concentrated by ultrafiltration using Microsep (PALL, Life Science) centrifugal device, and stored at -80°C.

### In vitro protein interaction experiments

Purified proteins were mixed together in pairwise combinations of untagged and his-tagged proteins. Before mixing, proteins in inclusion bodies were solubilised in denaturing buffer containing 6M guanidine-HCl, 20 mM Tris-HCl, pH 8, 10 mM MgCl_2_, 10 μM ZnCl_2_, 1 mM EDTA, 10mM DTT, 10% glycerol. Coupled proteins were mixed in 0.5-1 ml of denaturing buffer at equimolar ratio and adjusted to 0.2-0.5 mg/ml total protein concentration. Refolding of denatured molecules was achieved by removing guanidine-HCl from reaction mix through one-change dialysis against 500 ml of reconstitution buffer (20 mM Tris-HCl, pH 8, 0.2 M NaCl, 10 mM MgCl_2_, 10 μM ZnCl_2_, 0.25 mM EDTA, 0.5 mM DTT, 20% glycerol). Precipitate formed during dialysis was removed by centrifugation. Then supernatant was diluted 4-fold with start buffer (25 mM HEPES, pH 8.0, 0.5 M NaCl, 5% glycerol, 1 mM imidazole) and loaded on 0.5 ml His-Select Nickel Affinity Gel (Sigma) column equilibrated in the same buffer. The column was washed with start buffer, and proteins were eluted with three steps of start buffer containing 20, 100, 200 mM imidazole. Fractions were analyzed by SDS-PAGE and visualized by Coomassie-staining.

Reconstitution of α_2_β, and α_2_ββ' was performed as described above. The α_2 _β subassemblies were stepwise fractionated on Ni^2+^-affinity column with 10, 50, and 100 mM imidazole; α_2_ββ' RNAP core assembly reactions were fractionated by gel-filtration on a Superose 6 column (GE Healthcare) in the buffer containing 40 mM Tris-HCl, pH 8.0, 100 mM NaCl, 1 mM EDTA, and 1 mM 2-ME. Superose 6 fractions were checked for transcription activity on a nucleic acid scaffold. For this purpose, nucleic acid scaffold containing radioactively labelled 8-nt RNA primer (Figure 3A) was added into 10 μl of the target Superose 6 fraction to obtain artificial transcription elongation complexes and transcription was initiated by the addition of NTP and Mg^2+^. Reaction products were resolved by denaturing 20% PAGE and revealed by autoradiography.

### Bacterial two-hybrid assays

Gene fragments encoding *Francisella *α1NTD (residues 1-244), α2NTD (residues 1-242), α1CTD (residues 227-323), α2CTD (residues 226-318), were cloned in the plasmids pBRαLN and pACλcI to gain fusions with *E.coli *RNAP αNTD and bacteriophage λ cI proteins respectively [24,43,44]. FW102 F'O_L_2-62 reporter strain cells were co-transformed with every possible pairwise plasmid combination. Individual transformants were selected and grown in 2 ml of LB medium supplemented with 50 μg/ml carbenicillin, 25 μg/ml kanamycin, 25 μg/ml chloramphenicol, and 0.1 mM IPTG. β-galactosidase assays were performed as described earlier [43].

### RpoA comparative analysis

For comparative analysis of two RpoA paralogs in Francisella we retrieved the RefSeq database (NCBI) containing 1055 completely sequenced bacterial genomes on March 2010. A set of 368 RpoA sequences from 355 representative genomes was aligned with MUSCLE program [45], and the maximum likelihood tree for 246 informative aligned positions was built using FastTree program [46]. RAxML program [31] was used for reconstruction of the phylogenetic tree for γ-proteobacteria with β-proteobacteria as an outgroup. The same program was used for comparison of the maximum likelihood values for the best and constrained trees. The evolutionary model for tree reconstitution (WAG [47] with gamma-distributed evolutionary rates) was selected with ProtTest program [48].

## Authors' contributions

DM, KK, KS designed and interpreted the research; DM, KK performed molecular cloning, protein preparations, binding and transcription reactions and analyzed data obtained; KSM designed, performed and interpreted the bioinformatics study; the manuscript was prepared by KK, KSM, KS; all authors read and approved the final manuscript.

## Supplementary Material

Additional file 1 **Alpha alignment**. Click here for file

Additional file 2 **Constrained trees**. Click here for file

## References

[B1] Charity JC Costante-Hamm MM Balon EL Boyd DH Rubin EJ Dove SL Twin RNA polymerase-associated proteins control virulence gene expression in Francisella tularensis PLoS Pathog 2007 3 6 e84 10.1371/journal.ppat.0030084 17571921 PMC1891329

[B2] Zakharova N Hoffman PS Berg DE Severinov K The largest subunits of RNA polymerase from gastric helicobacters are tethered J Biol Chem 1998 273 31 19371 19374 10.1074/jbc.273.31.19371 9677352

[B3] Zakharova N Paster BJ Wesley I Dewhirst FE Berg DE Severinov KV Fused and overlapping rpoB and rpoC genes in Helicobacters, Campylobacters, and related bacteria J Bacteriol 1999 181 12 3857 3859 10368167 10.1128/jb.181.12.3857-3859.1999 PMC93870

[B4] Bergsland KJ Haselkorn R Evolutionary relationships among eubacteria, cyanobacteria, and chloroplasts: evidence from the rpoC1 gene of Anabaena sp. strain PCC 7120 J Bacteriol 1991 173 11 3446 3455 1904436 10.1128/jb.173.11.3446-3455.1991 PMC207958

[B5] Igarashi K Fujita N Ishihama A Identification of a subunit assembly domain in the alpha subunit of Escherichia coli RNA polymerase J Mol Biol 1991 218 1 1 6 10.1016/0022-2836(91)90865-4 2002495

[B6] Hayward RS Igarashi K Ishihama A Functional specialization within the alpha-subunit of Escherichia coli RNA polymerase J Mol Biol 1991 221 1 23 29 10.1016/0022-2836(91)80197-3 1920407

[B7] Blatter EE Ross W Tang H Gourse RL Ebright RH Domain organization of RNA polymerase alpha subunit: C-terminal 85 amino acids constitute a domain capable of dimerization and DNA binding Cell 1994 78 5 889 896 10.1016/S0092-8674(94)90682-3 8087855

[B8] Igarashi K Ishihama A Bipartite functional map of the E. coli RNA polymerase alpha subunit: involvement of the C-terminal region in transcription activation by cAMP-CRP Cell 1991 65 6 1015 1022 10.1016/0092-8674(91)90553-B 1646077

[B9] Ross W Gosink KK Salomon J Igarashi K Zou C Ishihama A Severinov K Gourse RL A third recognition element in bacterial promoters: DNA binding by the alpha subunit of RNA polymerase Science 1993 262 5138 1407 1413 10.1126/science.8248780 8248780

[B10] Ishihama A Protein-protein communication within the transcription apparatus J Bacteriol 1993 175 9 2483 2489 8478317 10.1128/jb.175.9.2483-2489.1993 PMC204548

[B11] Zillig W Palm P Heil A R Losick & M Chamberlin Function and Reassembly of Subunits of DNA-dependent RNA Polymerase RNA Polymerase 1976 6 Cold Spring Harbor, NY: Cold Spring Harbor Laboratory Press 101 125

[B12] Ishihama A Subunit of assembly of Escherichia coli RNA polymerase Adv Biophys 1981 14 1 35 7015808

[B13] Kimura M Fujita N Ishihama A Functional map of the alpha subunit of Escherichia coli RNA polymerase. Deletion analysis of the amino-terminal assembly domain J Mol Biol 1994 242 2 107 115 10.1006/jmbi.1994.1562 8089834

[B14] Heyduk T Heyduk E Severinov K Tang H Ebright RH Determinants of RNA polymerase alpha subunit for interaction with beta, beta', and sigma subunits: hydroxyl-radical protein footprinting Proc Natl Acad Sci USA 1996 93 19 10162 10166 10.1073/pnas.93.19.10162 8816769 PMC38354

[B15] Igarashi K Fujita N Ishihama A Sequence analysis of two temperature-sensitive mutations in the alpha subunit gene (rpoA) of Escherichia coli RNA polymerase Nucleic Acids Res 1990 18 20 5945 5948 10.1093/nar/18.20.5945 2235479 PMC332388

[B16] Kimura M Ishihama A Functional map of the alpha subunit of Escherichia coli RNA polymerase: amino acid substitution within the amino-terminal assembly domain J Mol Biol 1995 254 3 342 349 10.1006/jmbi.1995.0621 7490753

[B17] Kimura M Ishihama A Functional map of the alpha subunit of Escherichia coli RNA polymerase: insertion analysis of the amino-terminal assembly domain J Mol Biol 1995 248 4 756 767 10.1006/jmbi.1995.0258 7752238

[B18] Murakami K Kimura M Owens JT Meares CF Ishihama A The two alpha subunits of Escherichia coli RNA polymerase are asymmetrically arranged and contact different halves of the DNA upstream element Proc Natl Acad Sci USA 1997 94 5 1709 1714 10.1073/pnas.94.5.1709 9050843 PMC19981

[B19] Estrem ST Ross W Gaal T Chen ZW Niu W Ebright RH Gourse RL Bacterial promoter architecture: subsite structure of UP elements and interactions with the carboxy-terminal domain of the RNA polymerase alpha subunit Genes Dev 1999 13 16 2134 2147 10.1101/gad.13.16.2134 10465790 PMC316962

[B20] Cramer P Multisubunit RNA polymerases Curr Opin Struct Biol 2002 12 1 89 97 10.1016/S0959-440X(02)00294-4 11839495

[B21] Zhang G Darst SA Structure of the Escherichia coli RNA polymerase alpha subunit amino-terminal domain Science 1998 281 5374 262 266 9657722 10.1126/science.281.5374.262

[B22] Borukhov S Goldfarb A Recombinant Escherichia coli RNA polymerase: purification of individually overexpressed subunits and in vitro assembly Protein Expr Purif 1993 4 6 503 511 10.1006/prep.1993.1066 8286946

[B23] Ebright RH Busby S The Escherichia coli RNA polymerase alpha subunit: structure and function Curr Opin Genet Dev 1995 5 2 197 203 10.1016/0959-437X(95)80008-5 7613089

[B24] Dove SL Joung JK Hochschild A Activation of prokaryotic transcription through arbitrary protein-protein contacts Nature 1997 386 6625 627 630 10.1038/386627a0 9121589

[B25] Zalenskaya K Lee J Gujuluva CN Shin YK Slutsky M Goldfarb A Recombinant RNA polymerase: inducible overexpression, purification and assembly of Escherichia coli rpo gene products Gene 1990 89 1 7 12 10.1016/0378-1119(90)90199-2 2197183

[B26] Tang H Severinov K Goldfarb A Ebright RH Rapid RNA polymerase genetics: one-day, no-column preparation of reconstituted recombinant Escherichia coli RNA polymerase Proc Natl Acad Sci USA 1995 92 11 4902 4906 10.1073/pnas.92.11.4902 7761421 PMC41815

[B27] Korzheva N Mustaev A Kozlov M Malhotra A Nikiforov V Goldfarb A Darst SA A structural model of transcription elongation Science 2000 289 5479 619 625 10.1126/science.289.5479.619 10915625

[B28] Battistuzzi FU Feijao A Hedges SB A genomic timescale of prokaryote evolution: insights into the origin of methanogenesis, phototrophy, and the colonization of land BMC Evol Biol 2004 4 44 10.1186/1471-2148-4-44 15535883 PMC533871

[B29] Ciccarelli FD Doerks T von Mering C Creevey CJ Snel B Bork P Toward automatic reconstruction of a highly resolved tree of life Science 2006 311 5765 1283 1287 10.1126/science.1123061 16513982

[B30] Park HK Yoon JW Shin JW Kim JY Kim W rpoA is a useful gene for identification and classification of Streptococcus pneumoniae from the closely related viridans group streptococci FEMS Microbiol Lett 2010 305 1 58 64 10.1111/j.1574-6968.2010.01913.x 20158524

[B31] Stamatakis A RAxML-VI-HPC: maximum likelihood-based phylogenetic analyses with thousands of taxa and mixed models Bioinformatics 2006 22 21 2688 2690 10.1093/bioinformatics/btl446 16928733

[B32] Kannan N Chander P Ghosh P Vishveshwara S Chatterji D Stabilizing interactions in the dimer interface of alpha-subunit in Escherichia coli RNA polymerase: a graph spectral and point mutation study Protein Sci 2001 10 1 46 54 10.1110/ps.26201 11266593 PMC2249855

[B33] Lalo D Carles C Sentenac A Thuriaux P Interactions between three common subunits of yeast RNA polymerases I and III Proc Natl Acad Sci USA 1993 90 12 5524 5528 10.1073/pnas.90.12.5524 8516295 PMC46753

[B34] Larkin RM Guilfoyle TJ Reconstitution of yeast and Arabidopsis RNA polymerase alpha-like subunit heterodimers J Biol Chem 1997 272 19 12824 12830 10.1074/jbc.272.19.12824 9139743

[B35] Naryshkina T Rogulja D Golub L Severinov K Inter- and intrasubunit interactions during the formation of RNA polymerase assembly intermediate J Biol Chem 2000 275 40 31183 31190 10906130 10.1074/jbc.M003884200

[B36] Werner F Weinzierl RO A recombinant RNA polymerase II-like enzyme capable of promoter-specific transcription Mol Cell 2002 10 3 635 646 10.1016/S1097-2765(02)00629-9 12408830

[B37] Cramer P Bushnell DA Fu J Gnatt AL Maier-Davis B Thompson NE Burgess RR Edwards AM David PR Kornberg RD Architecture of RNA polymerase II and implications for the transcription mechanism Science 2000 288 5466 640 649 10.1126/science.288.5466.640 10784442

[B38] Werner F Grohmann D Evolution of multisubunit RNA polymerases in the three domains of life Nat Rev Microbiol 9 2 85 98 10.1038/nrmicro2507 21233849

[B39] Werner F Eloranta JJ Weinzierl RO Archaeal RNA polymerase subunits F and P are bona fide homologs of eukaryotic RPB4 and RPB12 Nucleic Acids Res 2000 28 21 4299 4305 10.1093/nar/28.21.4299 11058130 PMC113124

[B40] Lynch M Conery JS The evolutionary demography of duplicate genes J Struct Funct Genomics 2003 3 1-4 35 44 12836683

[B41] Deaconescu AM Chambers AL Smith AJ Nickels BE Hochschild A Savery NJ Darst SA Structural basis for bacterial transcription-coupled DNA repair Cell 2006 124 3 507 520 10.1016/j.cell.2005.11.045 16469698

[B42] Tang H Kim Y Severinov K Goldfarb A Ebright RH Escherichia coli RNA polymerase holoenzyme: rapid reconstitution from recombinant alpha, beta, beta', and sigma subunits Methods Enzymol 1996 273 130 134 8791605 10.1016/s0076-6879(96)73012-4

[B43] Dove SL Hochschild A A bacterial two-hybrid system based on transcription activation Methods Mol Biol 2004 261 231 246 15064462 10.1385/1-59259-762-9:231

[B44] Hu JC Kornacker MG Hochschild A Escherichia coli one- and two-hybrid systems for the analysis and identification of protein-protein interactions Methods 2000 20 1 80 94 10.1006/meth.1999.0908 10610807

[B45] Edgar RC MUSCLE: a multiple sequence alignment method with reduced time and space complexity BMC Bioinformatics 2004 5 113 10.1186/1471-2105-5-113 15318951 PMC517706

[B46] Price MN Dehal PS Arkin AP FastTree 2--approximately maximum-likelihood trees for large alignments PLoS One 2010 5 3 e9490 10.1371/journal.pone.0009490 20224823 PMC2835736

[B47] Whelan S Goldman N A general empirical model of protein evolution derived from multiple protein families using a maximum-likelihood approach Mol Biol Evol 2001 18 5 691 699 11319253 10.1093/oxfordjournals.molbev.a003851

[B48] Darriba D Taboada GL Doallo R Posada D ProtTest 3: fast selection of best-fit models of protein evolution Bioinformatics 2011 27 8 1164 1165 10.1093/bioinformatics/btr088 21335321 PMC5215816

