# Supplementary material for: Francisella RNA polymerase contains a heterodimer of non-identical α subunits
Source: BMC Mol Biol. 2011 Nov 22;12:50. doi: 10.1186/1471-2199-12-50 (PMC3294249; doi:10.1186/1471-2199-12-50)
Supplement: Additional file 1 — Alpha alignment. [file 1471-2199-12-50-S1.PDF]

MULTIPLE ALIGNMENT OF THE SELECTED αNTD SEQUENCES AND INTERFACE MAP OF THE α DIMERIZATION AND INTERACTION WITH β AND β' SUBUNITS.

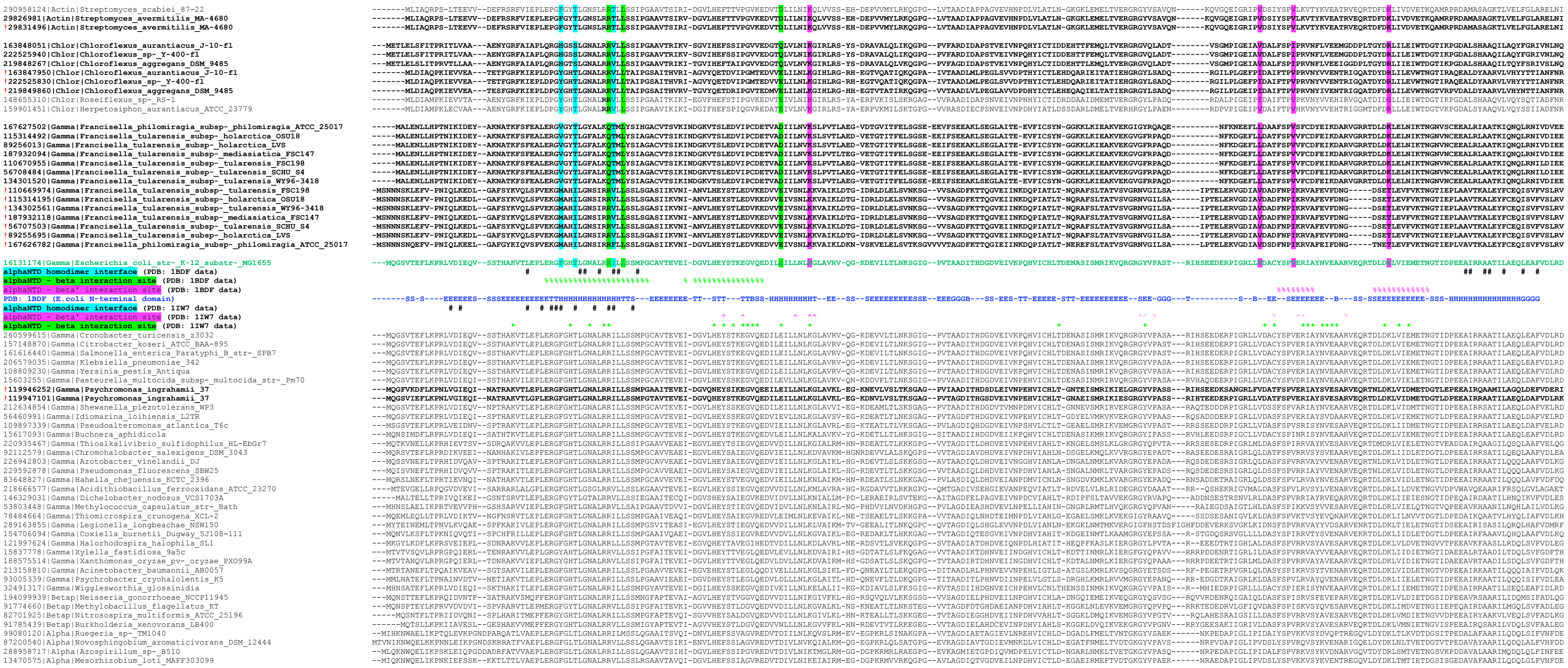

The MUSCLE program [1] was used for construction of multiple sequence alignment. Each sequence is labeled with GenBank Identifier (GI) number, five-letter taxonomy code and full systematic name of an organism.
The taxonomy code is the following: Gamma – γ-proteobacteria; Betap – β-proteobacteria; Alpha - α-proteobacteria; Actin – Actinobacteria; Chlor – Choloflexi. The "!" symbol indicates genes that are located in ribosomal operons.
The blue sequence corresponds to distribution of secondary structure elements in E. coli αNTD [2], named as G, H for 3-turn and 4-turn helix, respectively, T - short helix turn, E - extended β-sheets, B - β-bridge, S - bend.
The map of the interaction are shown according to PDB: 1BDF and PDB: 1IW7 and corresponding publications [2,3]. Color code corresponds to the interaction interface. Positions mutations in which cause defects in the respective interactions in *E.coli* are shaded by matching color [2,4,5].

1. Edgar RC: MUSCLE: multiple sequence alignment with high accuracy and high throughput. Nucleic Acids Res 2004, **32**: 1792-1797.
2. Zhang G, Darst SA: Structure of the *Escherichia coli* RNA polymerase alpha subunit amino-terminal domain. Science 1998, **281**(5374):262-266.
3. Vassylyev DG, Sekine S, Laptenko O, Lee J, Vassylyeva MN, Borukhov S, Yokoyama S: Crystal structure of a bacterial RNA polymerase holoenzyme at 2.6 A resolution. Nature 2002, **417**(6890):712-719.
4. Kimura M, Ishihama A: Functional map of the alpha subunit of *Escherichia coli* RNA polymerase: amino acid substitution within the amino-terminal assembly domain. *J Mol Biol* 1995, **254**(3):342-349.
5. Kannan N, Chander P, Ghosh P, Vishveshwara S, Chatterji D: Stabilizing interactions in the dimer interface of alpha-subunit in *Escherichia coli* RNA polymerase: a graph spectral and point mutation study. *Protein Sci* 2001, **10**(1):46-54.
