# Supplementary material for: Francisella RNA polymerase contains a heterodimer of non-identical α subunits
Source: BMC Mol Biol. 2011 Nov 22;12:50. doi: 10.1186/1471-2199-12-50 (PMC3294249; doi:10.1186/1471-2199-12-50)
Supplement: Additional file 2 — Constrained trees. [file 1471-2199-12-50-S2.PDF]

Constrained tree 1:
forsing Francisella RpoA1 and RpoA2 monophyly;

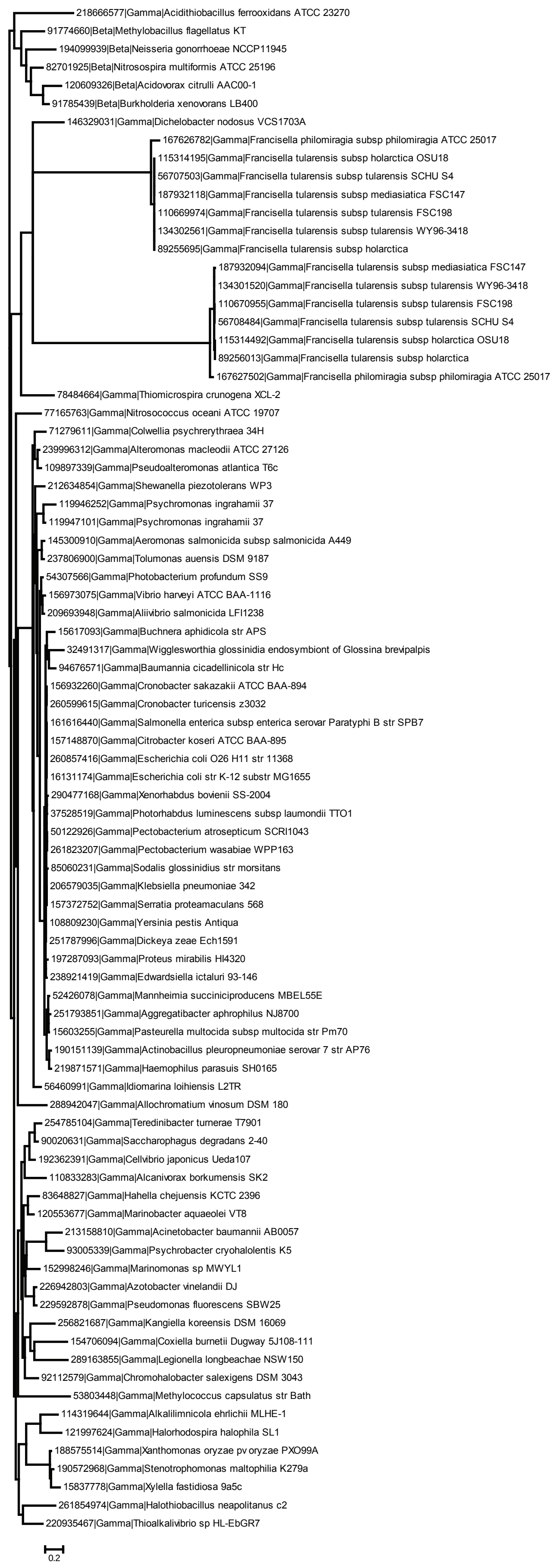

Constrained tree 2: forsing Francisella RpoA1 and RpoA2, Coxiella, Legionella and Thiomicrospira species monophyly;

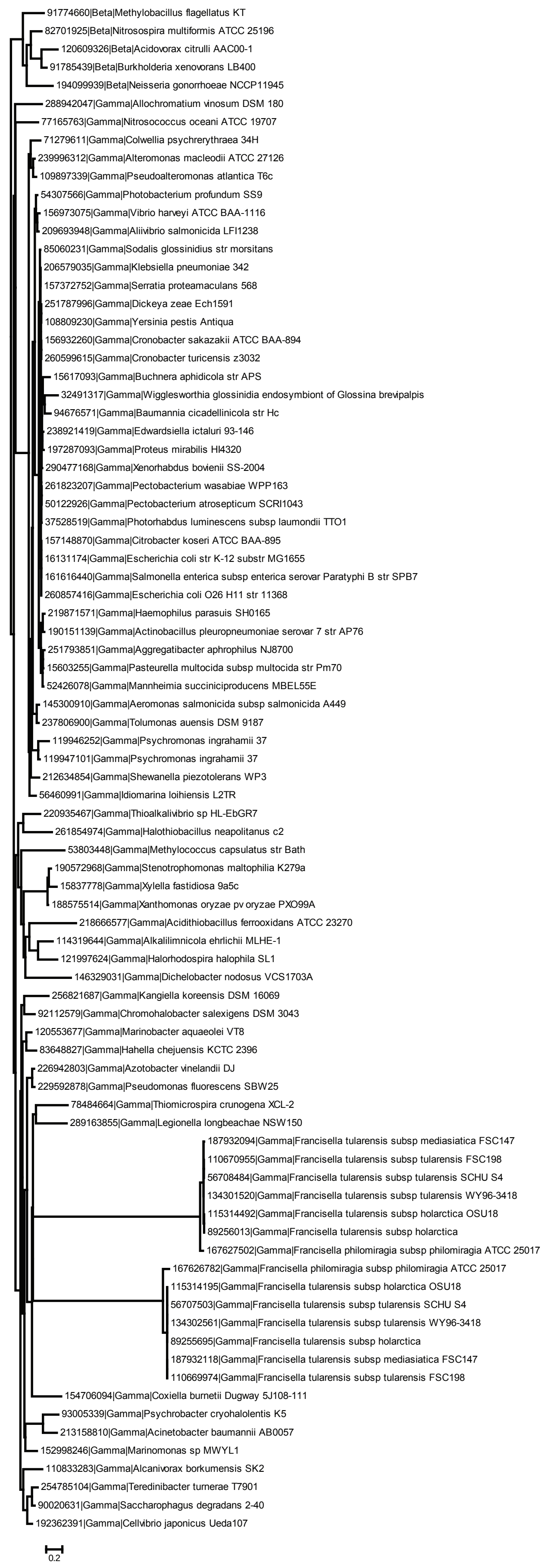

Model optimization, best Tree (Shown on the Figure 4B): -12523.140901
Constrained Tree: 1 Likelihood: -12524.016534 D(LH): -0.875633 SD: 14.078896 Significantly Worse: No
Constrained Tree: 2 Likelihood: -12533.081934 D(LH): -9.941033 SD: 27.558716 Significantly Worse: No
